# Epidemiological survey of patients with psoriatic arthritis in the Japanese Society for Psoriasis Research from 2017 to 2020

**DOI:** 10.1111/1346-8138.16603

**Published:** 2022-10-19

**Authors:** Koji Kamiya, Mamitaro Ohtsuki

**Affiliations:** ^1^ Department of Dermatology Jichi Medical University Shimotsuke Japan

**Keywords:** dermatology, epidemiology, Japan, Japanese Society for Psoriasis Research, psoriatic arthritis

## Abstract

The Japanese Society for Psoriasis Research (JSPR) conducted annual epidemiological surveys of patients with psoriatic arthritis (PsA). This study aimed to analyze a recent epidemiological survey of enrolled PsA patients in the JSPR from 2017 to 2020. A total of 1641 patients (1032 men [62.9%] and 609 women [37.1%]) were enrolled from 131 medical institutions. The mean ± standard deviation age of the patients was 51.4 ± 13.6 years and those at the onset of skin lesions and joint symptoms were 39.2 ± 15.8 and 47.9 ± 14.0 years, respectively. According to the Moll and Wright criteria, distal, oligoarticular, polyarticular, arthritis mutilans, and axial or spondyloarthritis types were 27.2%, 29.9%, 18.6%, 0.4%, and 6.6%, respectively. Approximately 56.3% of the patients had past history and comorbidities, such as hypertension (35.9%), dyslipidemia (20.7%), diabetes mellitus (19.2%), hyperuricemia (13.5%), cardiovascular disease (4.1%), and cerebrovascular disease (3.9%). Regarding systemic therapy, 55.9% and 45.5% of the patients were treated with oral medications and biologics, respectively. The most common oral medication was methotrexate (39.1%), followed by apremilast (27.4%). Non‐steroidal anti‐inflammatory drugs were also used in many patients (40.3%). Among the biologics, the most common was adalimumab (30.1%), followed by secukinumab (20.9%) and ixekizumab (17.0%). This survey shows the recent perspective of PsA in the Japanese society, which will lead to a better understanding of this disease, including patient characteristics, comorbidities, and treatment trends.

## 1 INTRODUCTION

Psoriasis is an immune‐mediated inflammatory condition, which is associated with many other diseases, most notably with psoriatic arthritis (PsA).^1^ The Japanese Society for Psoriasis Research (JSPR) has conducted annual epidemiological surveys of patients with psoriasis since 1982.^2^, ^3^, ^4^, ^5^ A total of 64 836 cases were registered and analyzed until 2018. While psoriasis and PsA share some pathogenetic and immunological features and their treatments frequently overlap, these diseases represent distinct genetic and immunological entities with differential therapeutic responsiveness.^1^ In Japan, Ohara et al.^6^ investigated the prevalence and clinical characteristics of PsA between March 2003 and February 2014. Yamamoto *et al*.^7^ also conducted the epidemiological survey of PsA patients from April 2014 to March 2015. Based on this survey, the characteristics of early and late‐onset PsA were also analyzed.^8^, ^9^ In 2016, the JSPR conducted a survey of the prevalence and current therapies of PsA in Japan.^10^ In 2017, the JSPR analyzed the switching of biologics for PsA treatment in Japan.^11^ Recently, Tsuruta et al.^12^ reported the prevalence of PsA in a prospective observational study of psoriasis between September 2019 and December 2020 from the Western Japan Psoriasis Registry (WJPR). However, there are not enough studies to examine the characteristics of PsA patients and treatment trends in Japan. Since 2017, the JSPR has conducted annual epidemiological surveys of PsA patients. This study aimed to analyze the recent epidemiological survey of enrolled PsA patients in the JSPR from 2017 to 2020.

## 2 METHODS

The JSPR partnered with medical institutions throughout Japan and used its own questionnaire to conduct annual surveys and collect data regarding newly diagnosed PsA cases (from April of the previous year to March of the survey year). A total of 131 medical institutions participated in the surveys for the present study, conducted from 2017 to 2020. The survey was designed to acquire information about patient characteristics, lifestyle habits, disease type and severity, family history, past history and comorbidities, exacerbating factors, focal infection, distribution of lesions, and current treatments. Only data from the completed surveys were included. This study was approved by the Ethical Committee of Jichi Medical University, the central institute that oversees the entire survey.

## 3 RESULTS

### 3.1 Patient characteristics

A total of 1641 patients (1032 men [62.9%] and 609 women [37.1%]) were enrolled from 2017 to 2020 (Table 1). The mean ± standard deviation (SD) height (cm) of the patients was 165.4 ± 9.1 (men, 170.2 ± 6.8; women, 157.1 ± 6.3 cm). Their mean ± SD weight (kg) was 69.1 ± 16.9 (men, 73.7 ± 16.1; women, 61.0 ± 15.2 kg). The mean ± SD body mass index (BMI) was 25.0 ± 5.2 (men, 25.3 ± 4.8; women, 24.6 ± 5.8). Some patients consumed alcohol (765 patients [46.6%]; 565 men [54.7%] and 200 women [32.8%]) and/or smoked (672 patients [41.0%]; 522 men [50.6%] and 150 women [24.6%]). Some patients had an atopic disposition (202 cases [12.3%]; 114 men [11.0%] and 88 women [14.4%]) and a history of visceral malignancies (79 cases [4.8%]; 40 men [3.9%] and 39 women [6.4%]). A total of 35 patients (2.1%; 18 men [1.7%] and 17 women [2.8%]) had a history of pustular psoriasis.

**TABLE 1:** Patient characteristics

	Male	Female	All
Number of patients	1032	609	1641
Height (cm), mean ± SD	170.2 ± 6.8	157.1 ± 6.3	165.4 ± 9.1
Weight (kg), mean ± SD	73.7 ± 16.1	61.0 ± 15.2	69.1 ± 16.9
BMI, mean ± SD	25.3 ± 4.8	24.6 ± 5.8	25.0 ± 5.2
Lifestyle habits			
Alcohol	565 (54.7%)	200 (32.8%)	765 (46.6%)
Smoking	522 (50.6%)	150 (24.6%)	672 (41.0%)
Past history			
Atopic disposition	114 (11.0%)	88 (14.4%)	202 (12.3%)
Malignancies	40 (3.9%)	39 (6.4%)	79 (4.8%)
Pustular psoriasis	18 (1.7%)	17 (2.8%)	35 (2.1%)

Abbreviations: BMI, body mass index; SD, standard deviation.

### 3.2 Population

**FIGURE 1:**
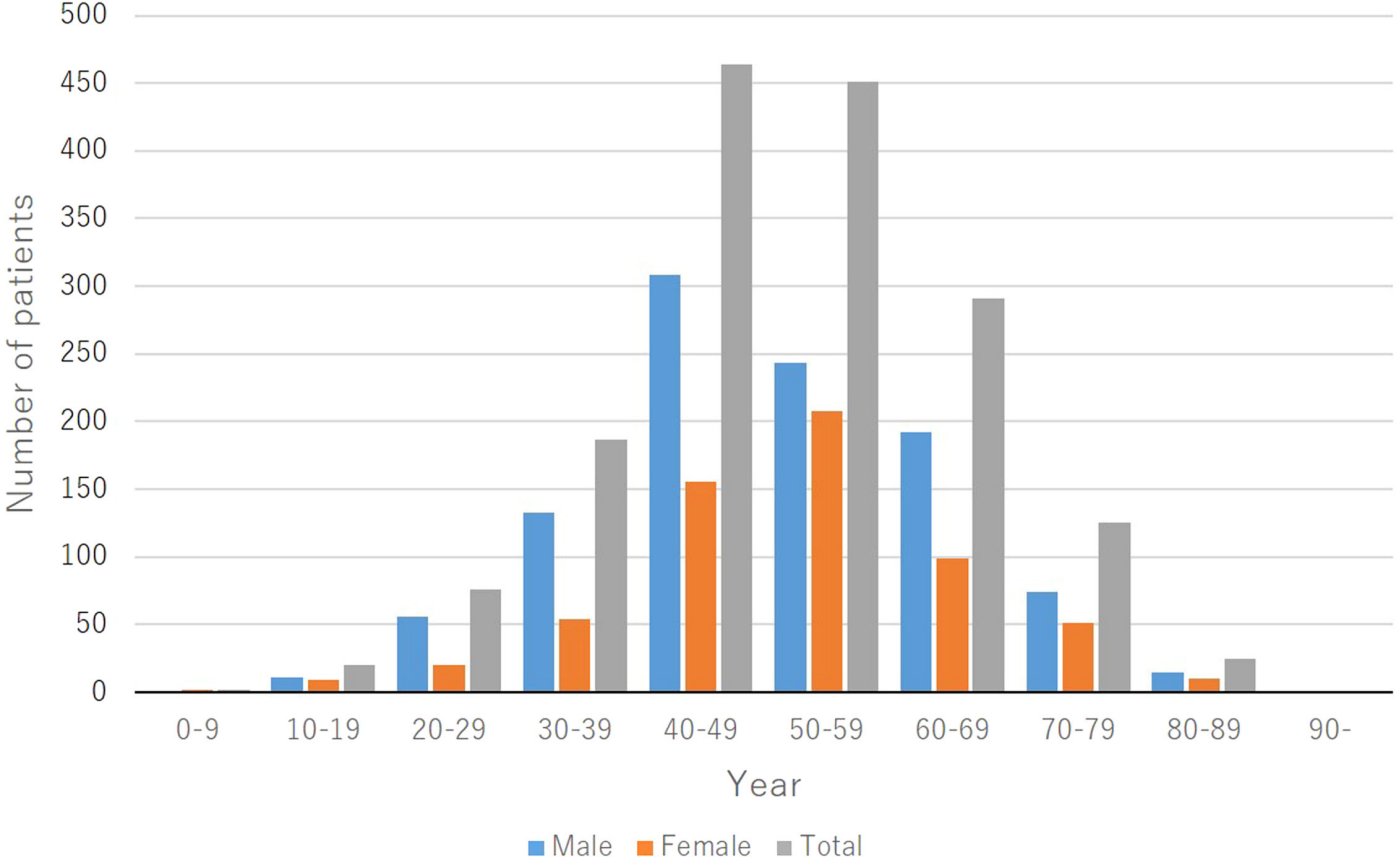
Age and sex distribution

The age at initial diagnosis varied from 8 to 89 years. The mean ± SD age of the patients was 51.4 ± 13.6 years (men, 50.7 ± 13.7 years; women, 52.5 ± 13.3 years). The age distributions were as follows: 2 patients aged 0–9 years (0.1%; 2 girls [0.3%]), 20 aged 10–19 years (1.2%; 11 boys [1.1%] and 9 girls [1.5%]), 76 aged 20–29 years (4.6%; 56 men [5.4%] and 20 women [3.3%]), 187 aged 30–39 years (11.4%; 133 men [12.9%] and 54 women [8.9%]), 464 aged 40–49 years (28.3%; 308 men [29.8%] and 156 women [25.6%]), 451 aged 50–59 years (27.5%; 243 men [23.5%] and 208 women [34.2%]), 291 aged 60–69 years (17.7%; 192 men [18.6%] and 99 women [16.3%]), 125 aged 70–79 years (7.6%; 74 men [7.2%] and 51 women [8.4%]), and 25 aged 80–89 years (1.5%; 15 men [1.5%] and 10 women [1.6%]), and none aged 90 years or older (Figure 1, Table S1).

### 3.3 Age at onset

**FIGURE 2:**
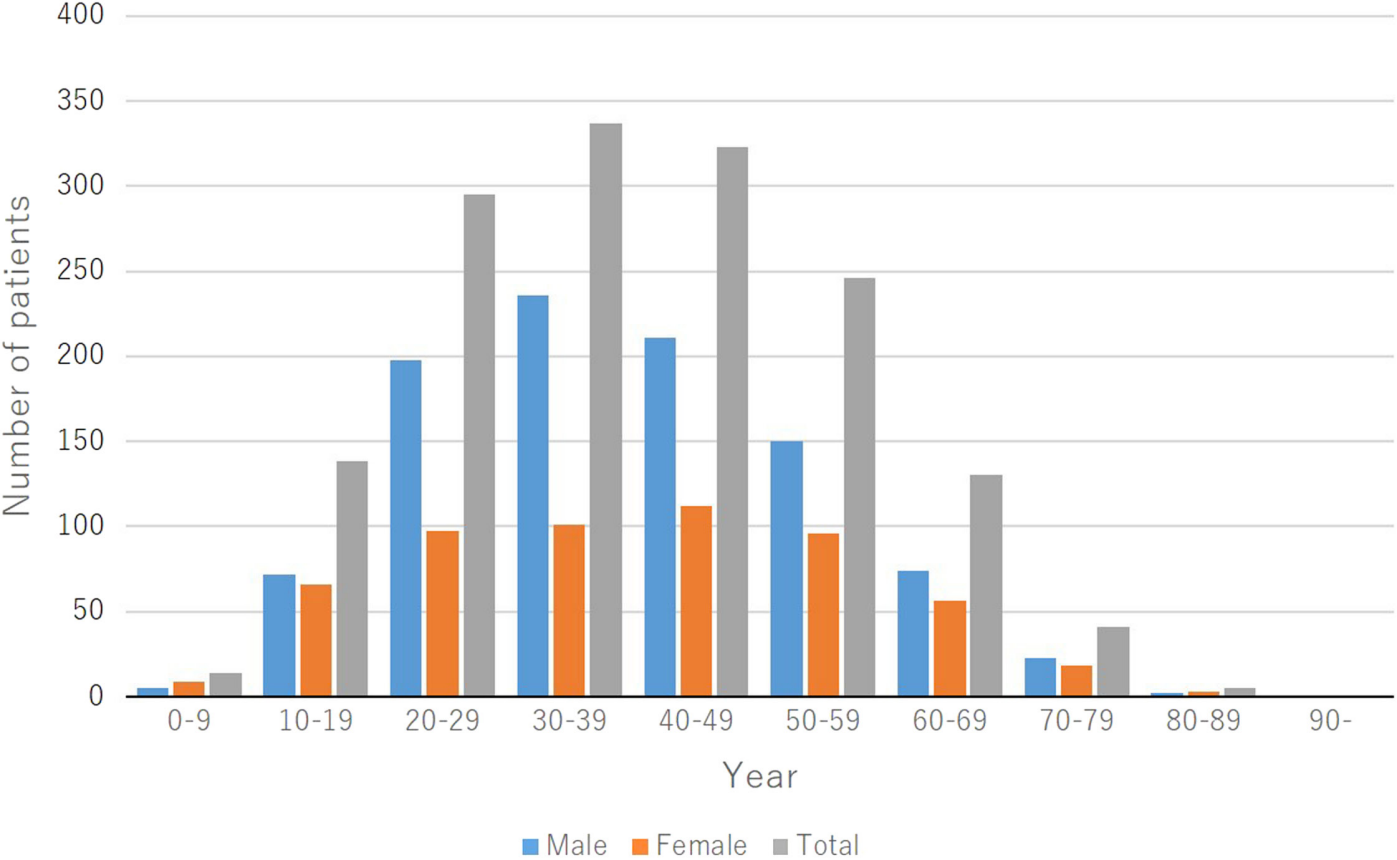
Age at onset of skin lesions

The mean ± SD age at the onset of skin lesions was 39.2 ± 15.8 years (men, 39.0 ± 14.9 years; women, 39.6 ± 17.3 years). The distributions of the onset age were as follows: 0–9 years for 14 patients (0.9%; 5 boys [0.5%] and 9 girls [1.6%]), 10–19 years for 138 patients (9.0%; 72 boys [7.4%] and 66 girls [11.8%]), 20–29 years for 295 patients (19.3%; 198 men [20.4%] and 97 women [17.4%]), 30–39 years for 337 patients (22.0%; 236 men [24.3%] and 101 women [18.1%]), 40–49 years for 323 patients (21.1%; 211 men [21.7%] and 112 women [20.1%]), 50–59 years for 246 patients (16.1%; 150 men [15.4%] and 96 women [17.2%]), 60–69 years for 130 patients (8.5%; 74 men [7.6%] and 56 women [10.0%]), 70–79 years for 41 patients (2.7%; 23 men [2.4%] and 18 women [3.2%]), 80–89 years for 5 patients (0.3%; 2 men [0.2%] and 3 women [0.5%]), and 90 years and older for no patients (Figure 2, Table S2).

**FIGURE 3:**
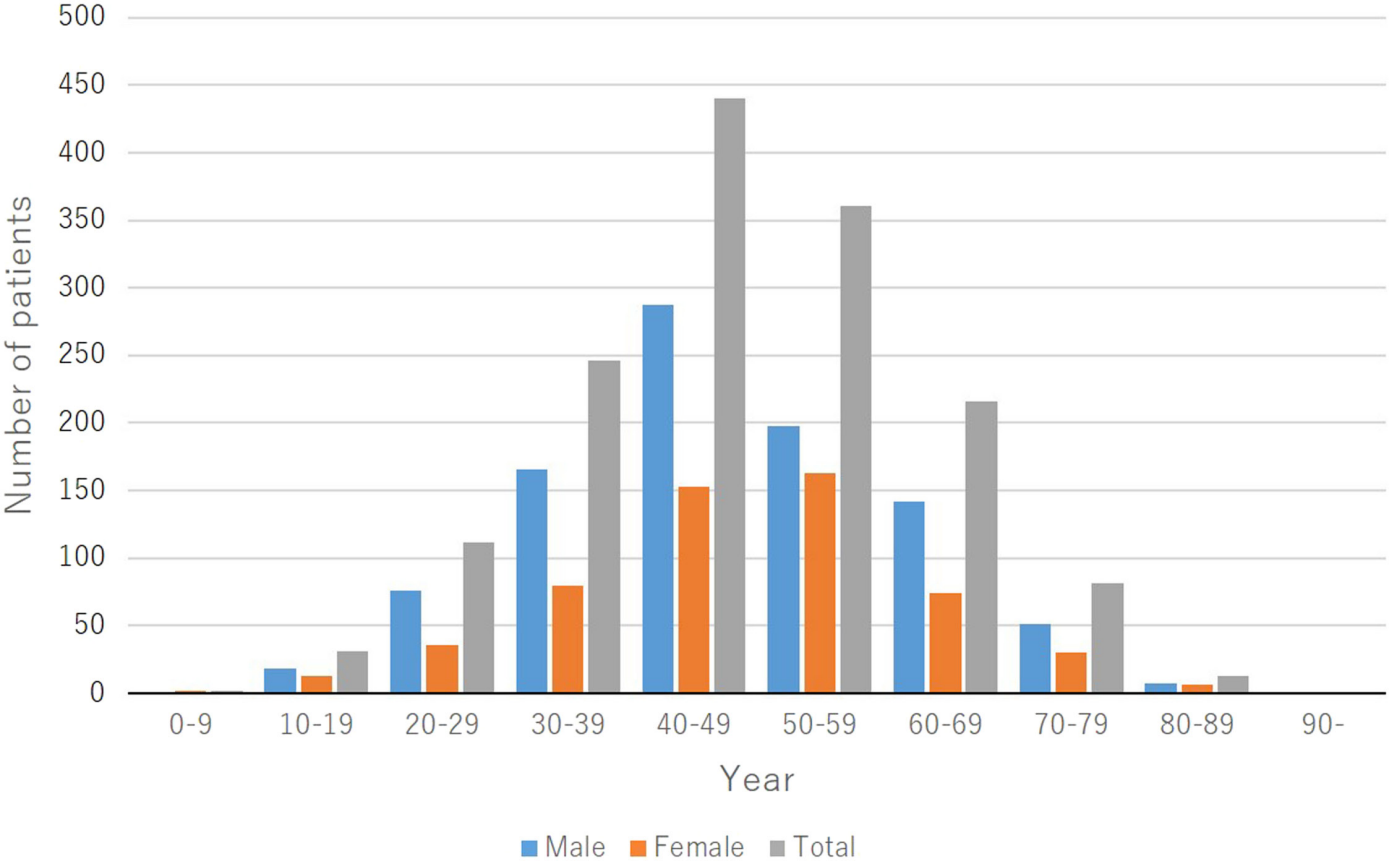
Age at onset of joint symptoms

The mean ± SD age at the onset of joint symptoms was 47.9 ± 14.0 years (men, 47.5 ± 14.0 years; women, 48.5 ± 14.0 years). The distributions of the onset age were as follows: 0–9 years for 2 patients (0.1%; 2 girls [0.4%]), 10–19 years for 31 patients (2.1%; 18 boys [1.9%] and 13 girls [2.3%]), 20–29 years for 112 patients (7.5%; 76 men [8.0%] and 36 women [6.5%]), 30–39 years for 246 patients (16.4%; 166 men [17.6%] and 80 women [14.4%]), 40–49 years for 440 patients (29.3%; 287 men [30.4%] and 153 women [27.5%]), 50–59 years for 361 patients (24.0%; 198 men [21.0%] and 163 women [29.3%]), 60–69 years for 216 patients (14.4%; 142 men [15.0%] and 74 women [13.3%]), 70–79 years for 81 patients (5.4%; 51 men [5.4%] and 30 women [5.4%]), 80–89 years for 13 patients (0.9%; 7 men [0.7%] and 6 women [1.1%]), and 90 years and older for no patients (Figure 3, Table S3).

### 3.4 Type and severity

A total of 1285 patients (78.3%) met the CASPAR criteria (832 men [80.6%] and 453 women [74.4%]). In the Moll and Wright criteria, the distal type, oligoarticular type, polyarticular type, arthritis mutilans type, and axial or spondyloarthritis type were 27.2% (men, 25.3%; women, 30.4%), 29.9% (men, 30.7%; women, 28.4%), 18.6% (men, 18.8%; women, 18.2%), 0.4% (men, 0.6%; women, 0%), and 6.6% (men, 7.4%; women, 5.4%), respectively (Table 2). The distributions of patients with <5%, 5%–10%, and >10% of the affected body surface area (BSA) were 47.7% (men, 44.1%; women, 53.9%), 23.6% (men, 24.2%; women, 22.5%), and 21.1% (men, 24.1%; women, 16.1%), respectively. The most common type of psoriasis was plaque psoriasis (91.4%; men, 92.1%; women: 90.3%). Itchiness was observed in 34.4% of the patients (men, 33.7%; women, 35.6%).

**TABLE 2:** Subtype and severity

	Male	Female	All
Subtype			
Distal	261 (25.3%)	185 (30.4%)	446 (27.2%)
Oligoarticular	317 (30.7%)	173 (28.4%)	490 (29.9%)
Polyarticular	194 (18.8%)	111 (18.2%)	305 (18.6%)
Arthritis mutilans	6 (0.6%)	0 (0%)	6 (0.4%)
Axial or spondyloarthritis	76 (7.4%)	33 (5.4%)	109 (6.6%)
Severity			
BSA <5%	455 (44.1%)	328 (53.9%)	783 (47.7%)
BSA 5%–10%	250 (24.2%)	137 (22.5%)	387 (23.6%)
BSA > 10%	249 (24.1%)	98 (16.1%)	347 (21.1%)
Unknown	78 (7.6%)	46 (7.6%)	124 (7.6%)

Abbreviation: BSA, body surface area.

### 3.5 Family history

A total of 108 patients (6.6%) had a family history of psoriasis (67 men [6.5%] and 41 women [6.7%]). The affected family members included fathers (19 men [43.3%] and 15 women [36.6%]), mothers (10 men [14.9%] and 7 women [17.1%]), children (5 men [7.5%] and 5 women [12.2%]), siblings (21 men [31.3%] and 8 women [19.5%]), and others (12 men [17.9%] and 9 women [22.0%]). In addition, 23 patients (1.4%) had a family history of PsA (9 men [0.9%] and 14 women [2.3%]). The affected family members included fathers (4 men [44.4%]), mothers (1 woman [7.1%]), children (2 women [14.3%]), siblings (1 man [11.1%] and 4 women [28.6%]), and others (4 men [44.4%] and 7 women [50.0%]). In contrast, 13 patients (0.8%) had a family history of palmoplantar pustulosis (6 men [0.6%] and 7 women [1.1%]). The affected family members included mothers (1 man [16.7%] and 1 woman [14.3%]), siblings (1 man [16.7%] and 1 woman [14.3%]), and others (3 men [50.0%] and 4 women [57.1%]).

### 3.6 Past history and comorbidities

In total, 903 patients (55.0%) had a history of comorbidities (581 men [56.3%] and 322 women [52.9%]). The patients' past history and comorbidities included hypertension (324 cases [35.9%]; 230 men [39.6%] and 94 women [29.2%]), dyslipidemia (187 cases [20.7%]; 133 men [22.9%] and 54 women [16.8%]), diabetes mellitus (173 cases [19.2%]; 125 men [21.5%] and 48 women [14.9%]), hyperuricemia (122 cases [13.5%]; 115 men [19.8%] and 7 women [2.2%]), cardiovascular disease (37 cases [4.1%]; 29 men [5.0%] and 8 women [2.5%]), and cerebrovascular disease (35 patients [3.9%]; 24 men [4.1%] and 11 women [3.4%]) (Table 3).

**TABLE 3:** Past history and comorbidities

	Male	Female	All
Hypertension	230 (39.6%)	94 (29.2%)	324 (35.9%)
Dyslipidemia	133 (22.9%)	54 (16.8%)	187 (20.7%)
Diabetes mellitus	125 (21.5%)	48 (14.9%)	173 (19.2%)
Hyperuricemia	115 (19.8%)	7 (2.2%)	122 (13.5%)
Cardiovascular disease	29 (5.0%)	8 (2.5%)	37 (4.1%)
Cerebrovascular disease	24 (4.1%)	11 (3.4%)	35 (3.9%)

### 3.7 Exacerbating factors

In total, 224 patients (13.7%) had exacerbating factors (133 men [12.9%] and 91 women [14.9%]). The exacerbating factors included stress (102 cases [45.5%]; 65 men [48.9%] and 37 women [40.7%]), season (70 cases [31.3%]; 48 men [36.1%] and 22 women [24.2%]), fatigue (36 cases [16.1%]; 25 men [18.8%] and 11 women [12.1%]), infection (22 cases [9.8%]; 12 men [9.0%] and 10 women [11.0%]), pregnancy (9 cases [4.0%]; 9 women [9.9%]), certain medications (7 cases [3.1%]; 3 men [2.3%] and 4 women [4.4%]), and sun exposure (6 cases [2.7%]; 4 men [3.0%] and 2 women [2.2%]) (Table 4). The percentages of patients with seasonal exacerbations that occurred in spring, summer, autumn, and winter were 11.4% (men, 14.6%; women, 4.5%), 21.4% (men, 22.9%; women, 18.2%), 12.9% (men, 14.6%; women, 9.1%), and 71.4% (men, 68.8%; women, 77.3%), respectively.

**TABLE 4:** Exacerbating factors

	Male	Female	All
Stress	65 (48.9%)	37 (40.7%)	102 (45.5%)
Seasonal factors	48 (36.1%)	22 (24.2%)	70 (31.3%)
Fatigue	25 (18.8%)	11 (12.1%)	36 (16.1%)
Infection	12 (9.0%)	10 (11.0%)	22 (9.8%)
Pregnancy	0 (0%)	9 (9.9%)	9 (4.0%)
Drug	3 (2.3%)	4 (4.4%)	7 (3.1%)
Sun exposure	4 (3.0%)	2 (2.2%)	6 (2.7%)

### 3.8 Focal infection

A total of 119 patients (7.3%) had some type of focal infections (71 men [6.9%] and 48 women [7.9%]). Focal infections included odontogenic infections (49 cases [41.2%]; 27 men [38.0%] and 22 women [45.8%]), tonsillitis (47 cases [39.5%]; 27 men [38.0%] and 20 women [41.7%]), sinusitis (22 cases [18.5%]; 13 men [18.3%] and 9 women [18.8%]), and otitis media (8 cases [6.7%]; 4 men [5.6%] and 4 women [8.3%]).

### 3.9 Distribution of skin lesions at the first examination

**FIGURE 4:**
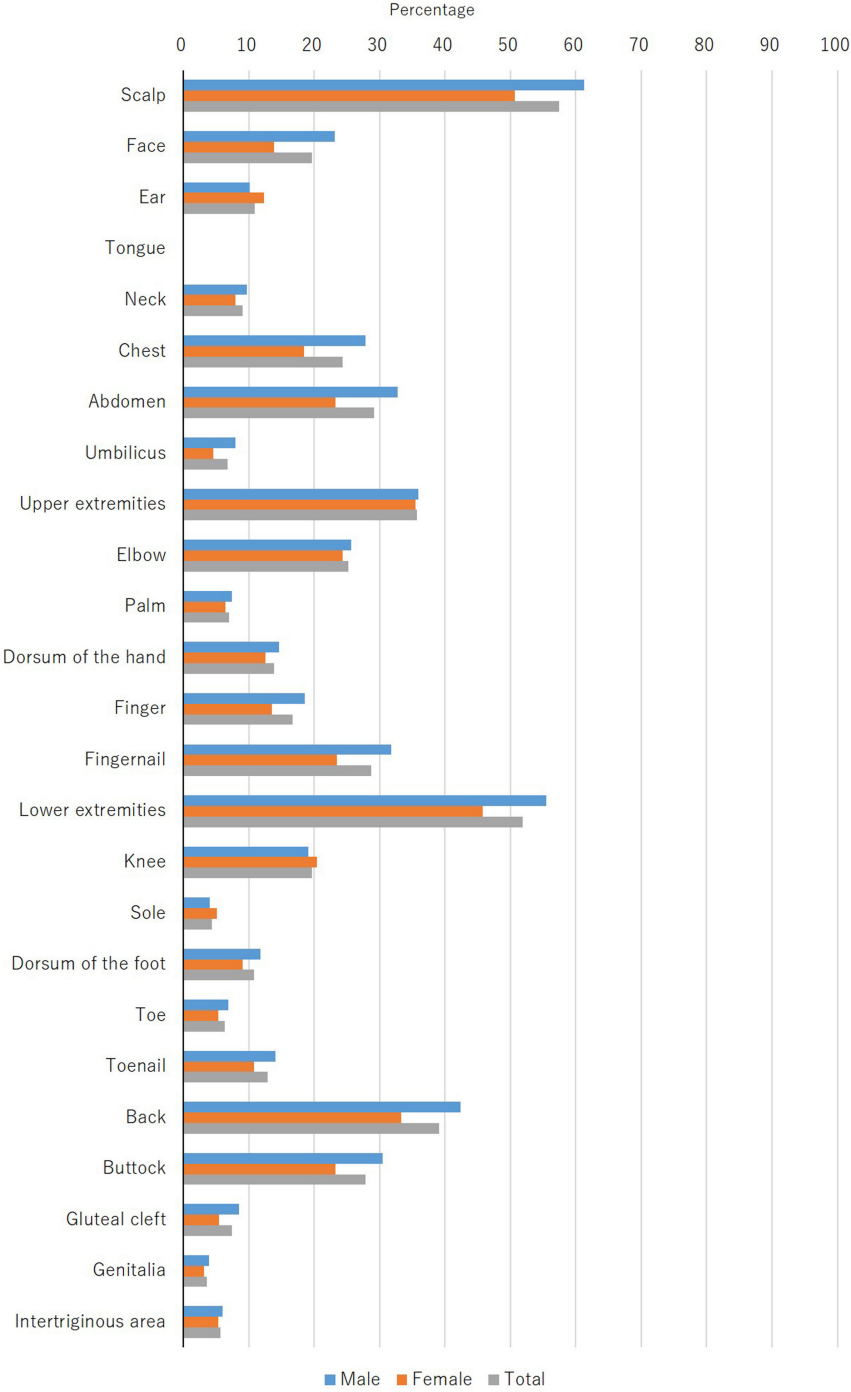
Anatomical distribution of skin lesions at the first examination

The skin lesions were located on the scalp (57.4%; men, 61.3%; women, 50.7%), the face (19.6%; men, 23.1%; women, 13.8%), the ear (10.9%; men, 10.1%; women, 12.3%), the tongue (0.1%; men, 0.1%; women, 0%), the neck (9.1%; men, 9.7%; women, 8.0%), the chest (24.3%; men, 27.8%; women, 18.4%), the abdomen (29.2%; men, 32.8%; women, 23.2%), the umbilicus (6.8%; men, 8.0%; women, 4.6%), the upper extremities (35.7%; men, 35.9%; women, 35.5%), the elbow (25.2%; men, 25.7%; women, 24.3%), the palm (7.0%; men, 7.4%; women, 6.4%), the dorsum of the hand (13.8%; men, 14.6%; women, 12.5%), the finger (16.7%; men, 18.6%; women, 13.5%), the fingernail (28.7%; men, 31.8% women, 23.5%), the lower extremities (51.9%; men, 55.5%; women, 45.8%), the knee (19.6%; men, 19.1%; women, 20.4%), the sole (4.4%; men, 4.0%; women, 5.1%), the dorsum of the foot (10.8%; men, 11.8%; women, 9.0%), the toe (6.3%; men, 6.9%; women, 5.3%), the toenail (12.9%; men, 14.1%; women, 10.8%), the back (39.1%; men, 42.4%; women, 33.3%), the buttocks (27.8%; men, 30.5%; women, 23.2%), the gluteal cleft (7.4%; men, 8.5%; women, 5.4%), the genitalia (3.6%; men, 3.9%; women, 3.1%), and the intertriginous area (5.7%; men, 6.0%; women, 5.3%) at the first examination (Figure 4, Table S4).

### 3.10 Distribution of joint symptoms at the first examination

**FIGURE 5:**
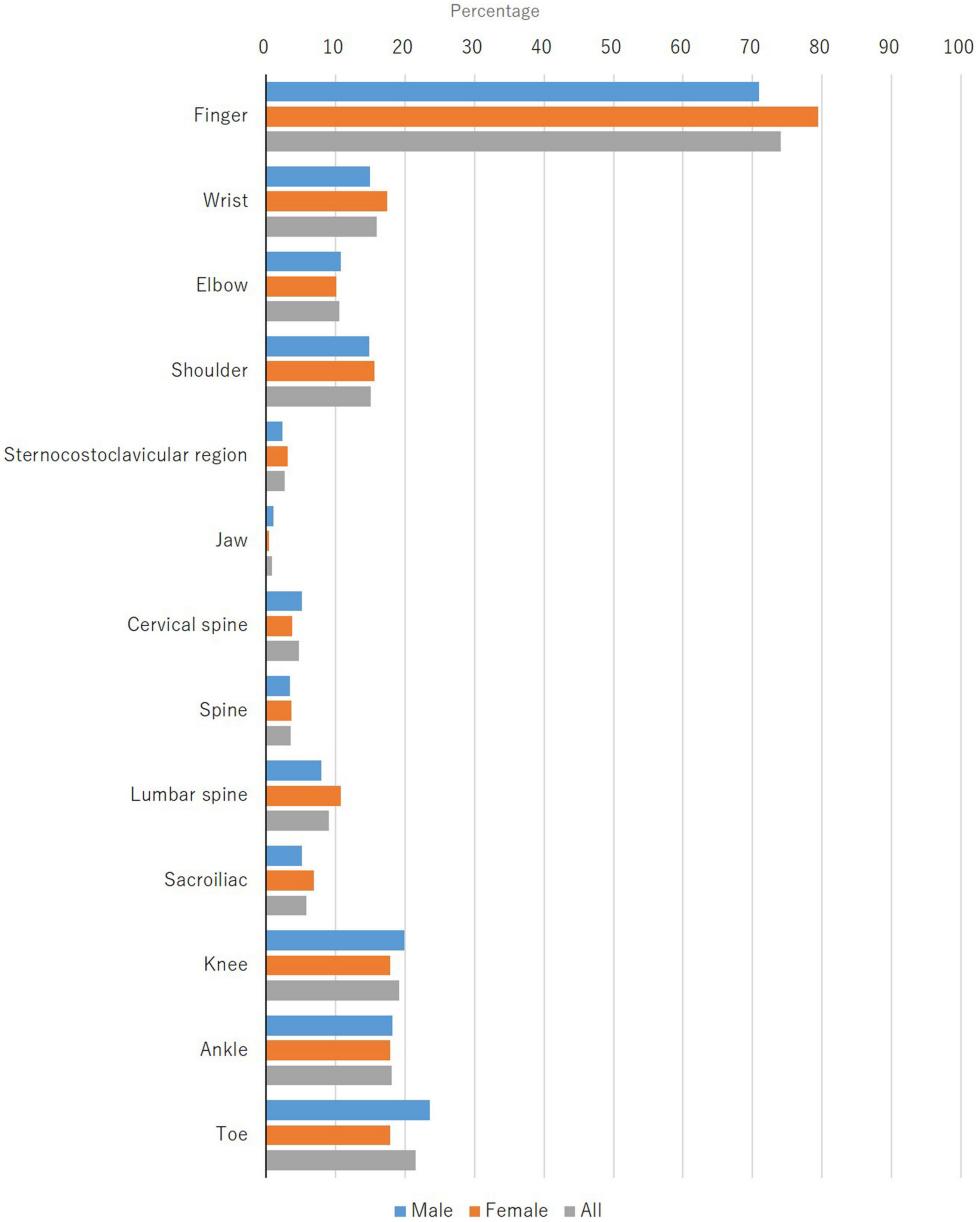
Anatomical distribution of joint tenderness at the first examination

The joint tenderness was located on the finger (74.1%; men, 70.9%; women, 79.4%), the wrist (15.9%; men, 15.0%; women, 17.4%), the elbow (10.5%; men, 10.7%; women, 10.1%), the shoulder (15.1%; men, 14.8%; women, 15.6%), the sternocostoclavicular region (2.7%; men, 2.4%; women, 3.1%), the jaw (0.8%; men, 1.1%; women, 0.4%), the cervical spine (4.7%; men, 5.2%; women, 3.8%), the spine (3.5%; men, 3.4%; women, 3.6%), the lumbar spine (9.0%; men, 8.0%; women, 10.7%), the sacroiliac (5.8%; men, 5.1%; women, 6.9%), the knee (19.1%; men, 19.9%; women, 17.9%), the ankle (18.1%; men, 18.2%; women, 17.9%), and the toe (21.5%; men, 23.6%; women, 17.9%) at the first examination (Figure 5, Table S5).

**FIGURE 6:**
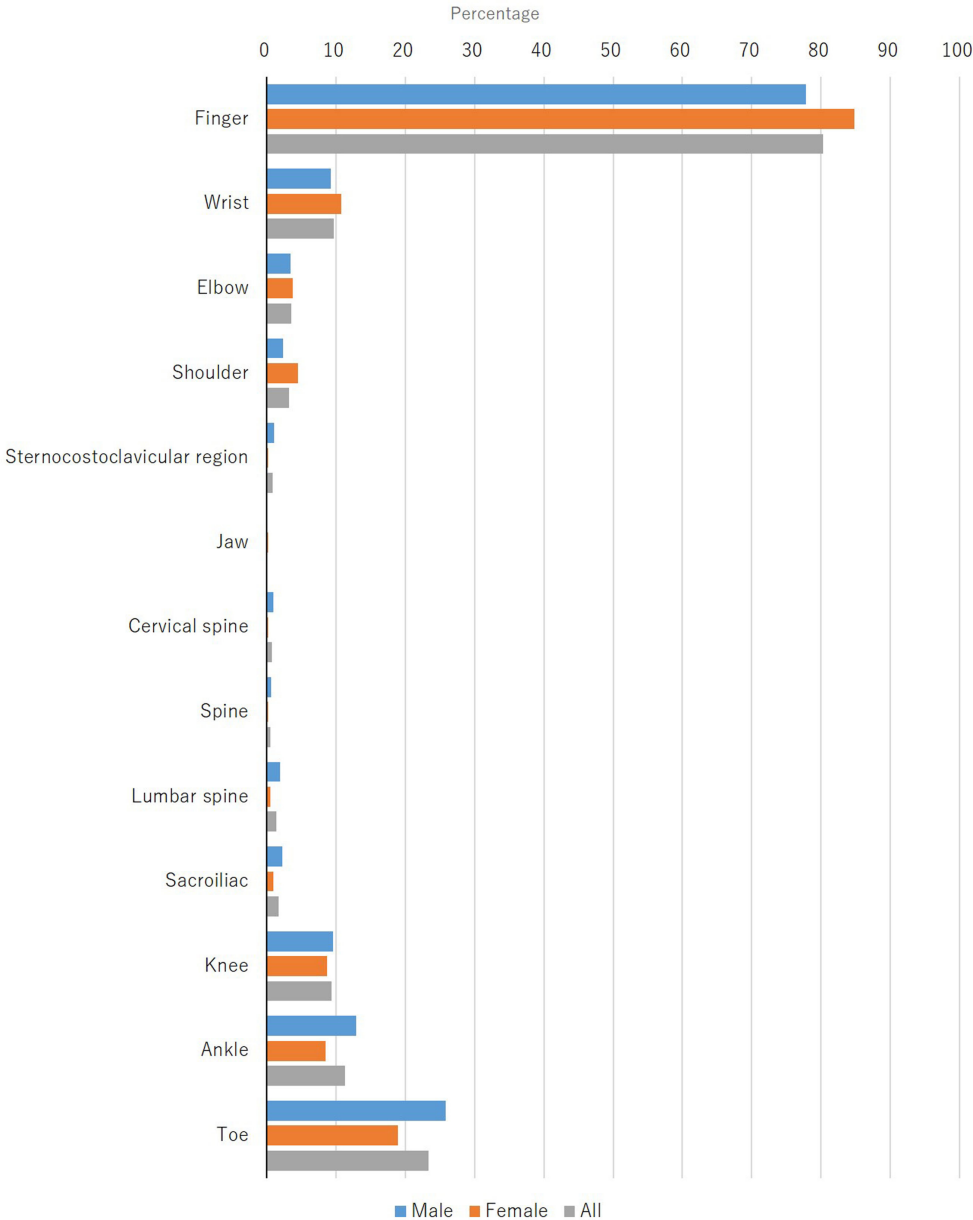
Anatomical distribution of joint swelling at the first examination

The joint swelling was located on the finger (80.3%; men, 77.8%; women, 84.8%), the wrist (9.7%; men, 9.2%; women, 10.7%), the elbow (3.5%; men, 3.4%; women, 3.7%), the shoulder (3.2%; men, 2.4%; women, 4.5%), the sternocostoclavicular region (0.8%; men, 1.1%; women, 0.2%), the jaw (0.1%; men, 0%; women, 0.2%), the cervical spine (0.7%; men, 1.0%; women, 0.2%), the spine (0.5%; men, 0.6%; women, 0.2%), the lumbar spine (1.4%; men, 1.9%; women, 0.5%), the sacroiliac (1.7%; men, 2.2%; women, 1.0%), the knee (9.3%; men, 9.6%; women, 8.7%), the ankle (11.3%; men, 12.9%; women, 8.5%), and the toe (23.3%; men, 25.8%; women, 18.9%) at the first examination (Figure 6, Table S6).

### 3.11 Treatments

**FIGURE 7:**
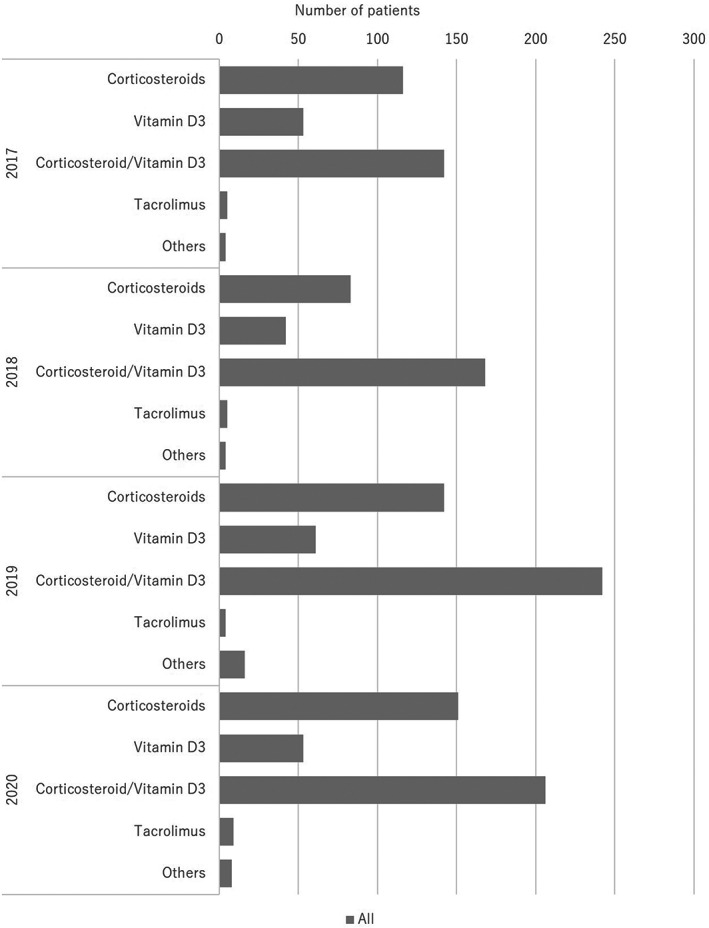
Treatment trends in topical therapy

**FIGURE 8:**
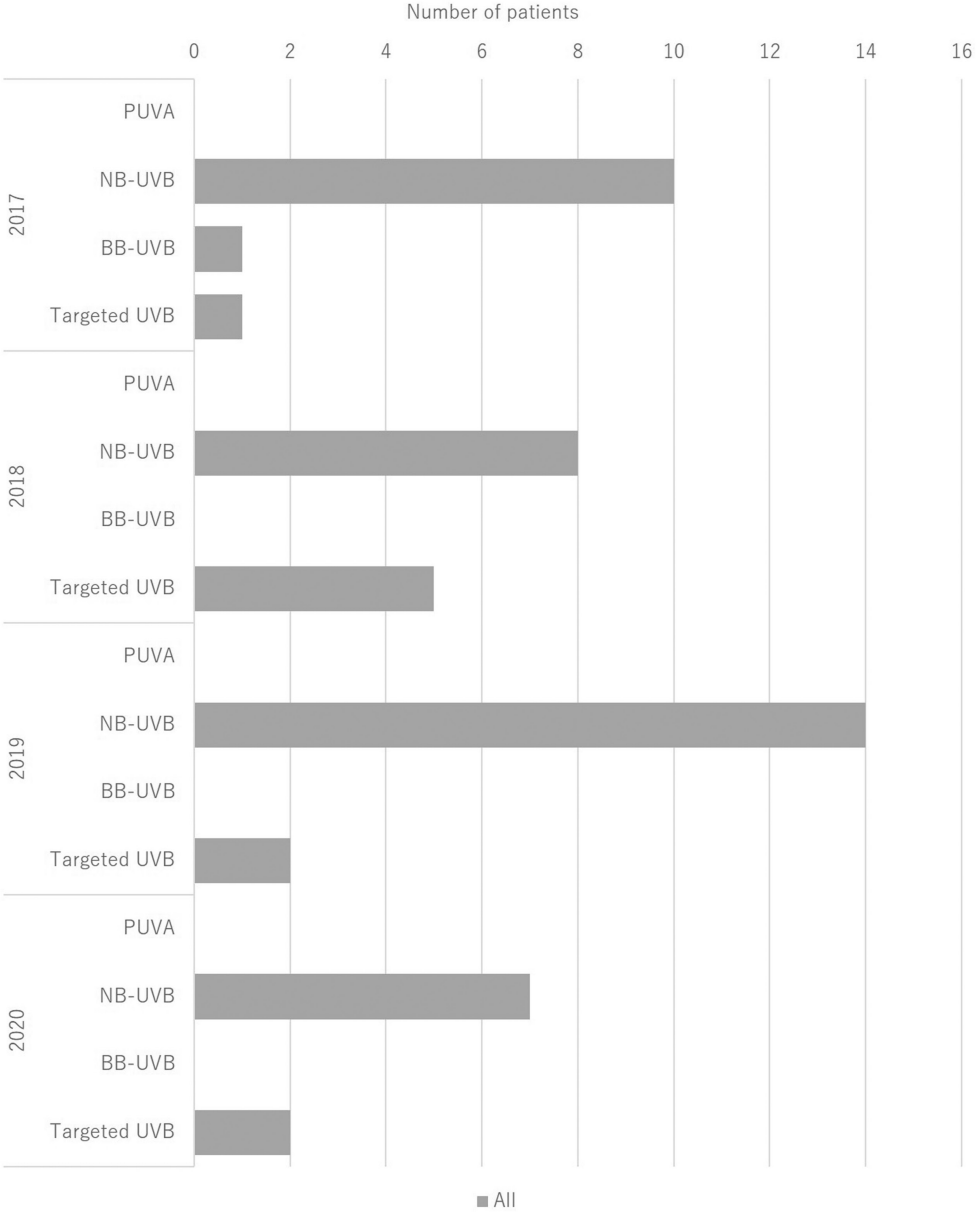
Treatment trends in phototherapy. BB‐UVB, broadband ultraviolet B; NB‐UVB, narrowband ultraviolet B; PUVA, psoralen ultraviolet A

**FIGURE 9:**
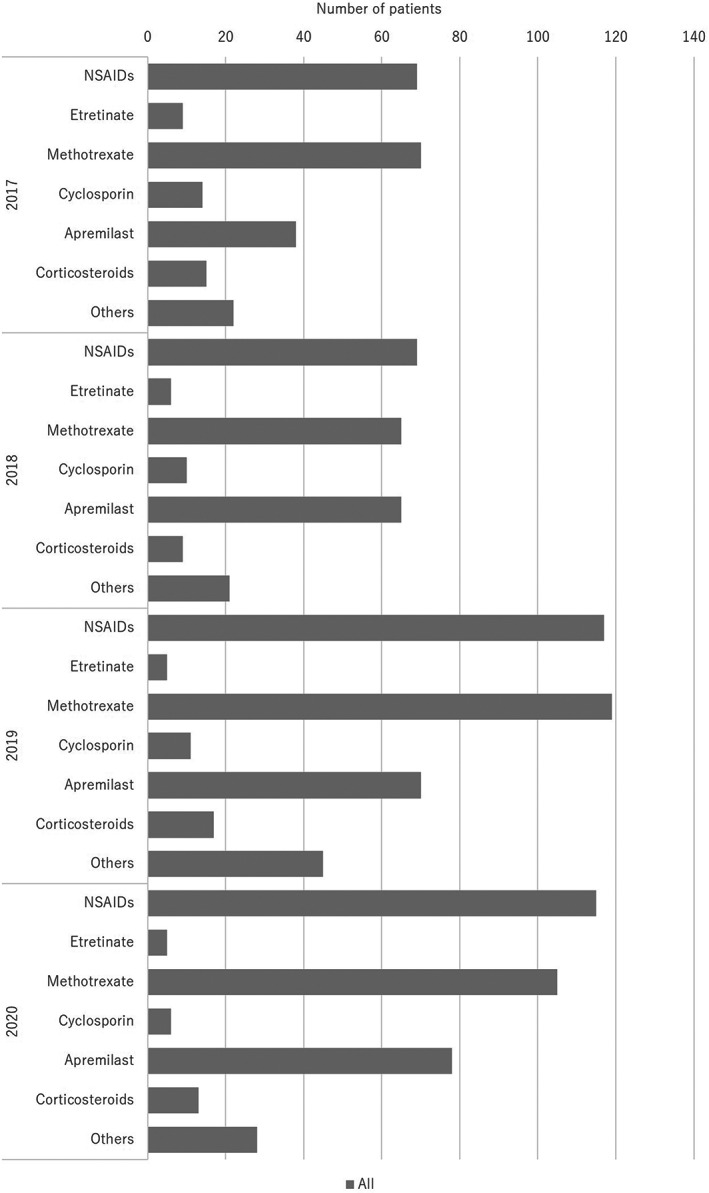
Treatment trends in the oral medication. NSAIDs, non‐steroidal anti‐inflammatory drugs

**FIGURE 10:**
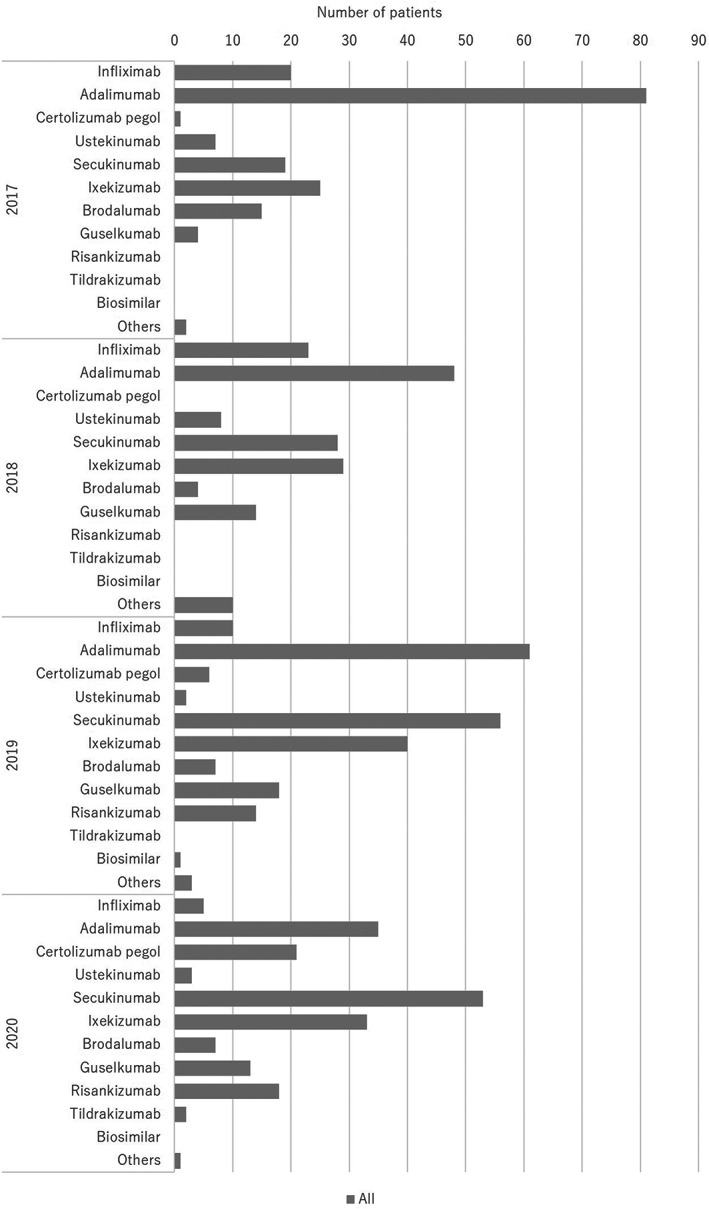
Treatment trends in the biologics

The treatments are summarized in Table 5 and the treatment trends are shown in Figures 7, 8, 9, 10 and Tables S7–S10. A total of 1126 patients (68.6%) received topical therapy (698 men [67.6%] and 428 women [70.3%]). Topical therapy included corticosteroids (492 patients [43.7%]; 302 men [43.3%] and 190 women [44.4%]), vitamin D_3_ (209 patients [18.6%]; 138 men [19.8%] and 71 women [16.6%]), corticosteroid/vitamin D_3_ combinations (758 patients [67.3%]; 470 men [67.3%] and 288 women [67.3%]), tacrolimus (23 patients [2.0%]; 20 men [2.9%] and 3 women [0.7%]), and others (32 patients [2.8%]; 18 men [2.6%] and 14 women [3.3%]).

**TABLE 5:** Treatments for psoriasis

	Male	Female	All
Topical therapy	698 (67.6%)	428 (70.3%)	1126 (68.6%)
Corticosteroids	302 (43.3%)	190 (44.4%)	492 (43.7%)
Vitamin D_3_	138 (19.8%)	71 (16.6%)	209 (18.6%)
Corticosteroid/vitamin D_3_	470 (67.3%)	288 (67.3%)	758 (67.3%)
Tacrolimus	20 (2.9%)	3 (0.7%)	23 (2.0%)
Others	18 (2.6%)	14 (3.3%)	32 (2.8%)
Phototherapy	29 (2.8%)	21 (3.4%)	50 (3.0%)
PUVA	0 (0%)	0 (0%)	0 (0%)
NB‐UVB	22 (75.9%)	17 (81.0%)	39 (78.0%)
BB‐UVB	1 (3.4%)	0 (0%)	1 (2.0%)
Targeted UVB	6 (20.7%)	4 (19.0%)	10 (20.0%)
Systemic therapy			
Oral medication	558 (54.1%)	359 (58.9%)	917 (55.9%)
Etretinate	18 (3.2%)	7 (1.9%)	25 (2.7%)
Methotrexate	232 (41.6%)	127 (35.4%)	359 (39.1%)
Cyclosporin	24 (4.3%)	17 (4.7%)	41 (4.5%)
Apremilast	151 (27.1%)	100 (27.9%)	251 (27.4%)
Corticosteroids	36 (6.5%)	18 (5.0%)	54 (5.9%)
NSAIDs	216 (38.7%)	154 (42.9%)	370 (40.3%)
Others	67 (12.0%)	49 (13.6%)	116 (12.6%)
Biologics	479 (46.4%)	268 (44.0%)	747 (45.5%)
Infliximab	47 (9.8%)	11 (4.1%)	58 (7.8%)
Adalimumab	141 (29.4%)	84 (31.3%)	225 (30.1%)
Certolizumab pegol	20 (4.2%)	8 (3.0%)	28 (3.7%)
Ustekinumab	10 (2.1%)	10 (3.7%)	20 (2.7%)
Secukinumab	96 (20.0%)	60 (22.4%)	156 (20.9%)
Ixekizumab	85 (17.7%)	42 (15.7%)	127 (17.0%)
Brodalumab	22 (4.6%)	11 (4.1%)	33 (4.4%)
Guselkumab	24 (5.0%)	25 (9.3%)	49 (6.6%)
Risankizumab	24 (5.0%)	8 (3.0%)	32 (4.3%)
Tildrakizumab	1 (0.2%)	1 (0.4%)	2 (0.3%)
Biosimilar	1 (0.2%)	0 (0%)	1 (0.1%)
Others	8 (1.7%)	8 (3.0%)	16 (2.1%)
GMA	1 (0.1%)	1 (0.2%)	2 (0.1%)

Abbreviations: BB‐UVB, broadband ultraviolet B; GMA, granulocyte and monocyte adsorptive apheresis; NB‐UVB, narrowband ultraviolet B; NSAIDs, non‐steroidal anti‐inflammatory drugs; PUVA, psoralen ultraviolet A.

Fifty patients (3.0%) received phototherapy (29 men [2.8%] and 21 women [3.4%]). Phototherapy included narrowband (NB)‐ ultraviolet B (UVB) (39 patients [78.0%]; 22 men [75.9%] and 17 women [81.0%]), broadband (BB)‐UVB (1 patient [2.0%]; 1 man [3.4%]), and targeted UVB (10 patients [20.0%]; 6 men [20.7%] and 4 women [19.0%]). No patients were treated with psoralen ultraviolet A (PUVA).

Systemic therapy could be divided into oral medications and biologics. A total of 917 patients (55.9%) were treated with oral medications (558 men [54.1%] and 359 women [58.9%]). Oral medications included etretinate (25 cases [2.7%]; 18 men [3.2%] and 7 women [1.9%]), methotrexate (359 cases [39.1%]; 232 men [41.6%] and 127 women [35.4%]), cyclosporin (41 cases [4.5%]; 24 men [4.3%] and 17 women [4.7%]), apremilast (251 cases [27.4%]; 151 men [27.1%] and 100 women [27.9%]), corticosteroids (54 cases [5.9%]; 36 men [6.5%] and 18 women [5.0%]), non‐steroidal anti‐inflammatory drugs (NSAIDs) (370 cases [40.3%]; 216 men [38.7%] and 154 women [42.9%]), and others (116 cases [12.6%]; 67 men [12.0%] and 49 women [13.6%]).

In total, 747 patients (45.5%) were treated with biologics (479 men [46.4%] and 268 women [44.0%]). The biologics included infliximab (58 cases [7.8%]; 47 men [9.8%] and 11 women [4.1%]), adalimumab (225 cases [30.1%]; 141 men [29.4%] and 84 women [31.3%]), certolizumab pegol (28 cases [3.7%]; 20 men [4.2%] and 8 women [3.0%]), ustekinumab (20 cases [2.7%]; 10 men [2.1%] and 10 women [3.7%]), secukinumab (156 cases [20.9%]; 96 men [20.0%] and 60 women [22.4%]), ixekizumab (127 cases [17.0%]; 85 men [17.7%] and 42 women [15.7%]), brodalumab (33 cases [4.4%]; 22 men [4.6%] and 11 women [4.1%]), guselkumab (49 cases [6.6%]; 24 men [5.0%] and 25 women [9.3%]), risankizumab (32 cases [4.3%]; 24 men [5.0%] and 8 women [3.0%]), tildrakizumab (2 cases [0.3%]; 1 man [0.2%] and 1 woman [0.4%]), biosimilars (1 case [0.1%]; 1 man [0.2%]), and others (16 cases [2.1%]; 8 men [1.7%] and 8 women [3.0%]). Of these, approximately 13.5% of patients presented with paradoxical reactions. Discontinuation or bioswitching was observed in 99 patients. In patients who received bioswitching, the most common initial biologics were adalimumab (28 cases), followed by infliximab (20 cases), secukinumab (16 cases), ixekizumab (8 cases), and brodalumab (7 cases). In patients initially treated with adalimumab, the second most common biologics were secukinumab (9 cases), ixekizumab (7 cases), brodalumab (6 cases), infliximab (3 cases), and brodalumab (3 cases). In patients initially treated with infliximab, the second most common biologics were adalimumab (9 cases), secukinumab (3 cases), ixekizumab (3 cases), risankizumab (2 cases), ustekinumab (2 cases), and guselkumab (1 case). In patients initially treated with secukinumab, the second most common biologics were infliximab (5 cases), brodalumab (3 cases), certolizumab pegol (3 cases), adalimumab (2 cases), guselkumab (2 cases), and ustekinumab (1 case). In patients initially treated with ixekizumab, the second most common biologics were adalimumab (3 cases), infliximab (2 cases), certolizumab pegol (1 case), secukinumab (1 case), and guselkumab (1 case). In patients initially treated with brodalumab, the second most common biologics were adalimumab (3 cases), ixekizumab (2 cases), infliximab (1 case), and guselkumab (1 case). A total of 21 cases were treated with more than three biologics. The total number of biologics used was 3 (15 cases), 4 (5 cases), and 6 (1 case), respectively. Among these patients, the initial biologics were infliximab (4 cases), ustekinumab (4 cases), secukinumab (4 cases), and adalimumab (3 cases). Granulocyte and monocyte adsorption apheresis (GMA), approved for the treatment of PsA in 2019, was used to treat PsA in only 2 patients ([0.1%]; 1 man [0.1%] and 1 woman [0.2%]).

## 4 DISCUSSION

In the present survey, a total of 1641 PsA cases (1032 men [62.9%] and 609 women [37.1%]) were enrolled during 2017–2020 from 131 medical institutions. In the past JSPR survey from 2014 to 2015, a total of 1282 PsA cases were enrolled with the male:female ratio of 1.9.^7^ In the WJPR, a total of 379 PsA cases (268 men and 111 women) were enrolled.^12^ In another study by rheumatologists in Japan, men accounted for 59.9% of a total of 431 PsA cases.^6^ Although the male or female predominance varies among different countries,^13^ the male predominance is a distinctive feature in Japanese patients with PsA.

In terms of age distribution, the proportion of patients increased gradually after the age of 20 years, peaked in the age groups of 40–49 and 50–59 years, and then gradually decreased (Figure 1). In men, the proportion increased gradually after the age of 20 years, peaked in the age group of 40–49 years, and then gradually decreased. In women, the proportion increased gradually, and peaked in the age group of 50–59 years. The peak age of men was lower than that of women. In the past JSPR survey from 2014 to 2015, the proportion of patients with PsA increased after the age of 20 years, peaked in the age group of 40–49 years, and then gradually decreased.^8^ The age distribution of the present survey was similar to that of the past JSPR survey.

Regarding the age at the onset of skin lesions, the majority of patients were in the age groups of 30–39 years (22.0%) and 40–49 years (21.1%), followed by the age groups of 20–29 years (19.3%) and 50–59 years (16.1%) (Figure 2). Most men had an age at onset of 30–39 years (24.3%), followed by 40–49 years (21.7%) and 20–29 years (20.4%), while women were equally distributed among the onset age groups of 20–29 years, 30–39 years, 40–49 years, and 50–59 years. In contrast, regarding the age at the onset of joint symptoms, the majority of patients were in the age of 40–49 years (29.3%), followed by the age group of 50–59 years (24.0%) (Figure 3). Most men had an age of onset of 40–49 years (30.4%), followed by 50–59 years (21.0%), while most women had an age of onset of 50–59 years (29.3%), followed by 40–49 years (27.5%). These findings suggested that skin lesions preceded the joint symptoms in most patients. In the age group of 0–9 years, there were more girls than boys, and there were no boys with joint symptoms.

In the Moll and Wright criteria, the polyarticular type was the most common (36%), followed by the distal type (26%) and oligoarthritis type (22%) in the past JSPR survey of PsA from 2014 to 2015.^7^ In the present survey, the most common type was the oligoarticular type (29.9%), followed by the distal type (27.2%) and polyarticular type (18.6%). The oligoarticular type was the most common in men (30.7%), whereas the distal type was the most common in women (30.4%). Regarding severity, patients with <5%, 5%–10%, and >10% of the affected BSA were 47.7% (men, 44.1%; women, 53.9%), 23.6% (men, 24.2%; women, 22.5%), and 21.1% (men, 24.1%; women, 16.1%), respectively. PsA patients, particularly women, tended to have milder skin symptoms, compared with the characteristics observed in the past JSPR survey of psoriasis.^5^ The most common type of psoriasis was plaque psoriasis (91.4%) in the present survey, which was also confirmed by other past surveys.^7^, ^12^

A family history of psoriasis is often observed, and past JSPR surveys revealed that approximately 4.4%–6.4% of the patients with psoriasis had a family history, with the father as the most commonly affected family member.^2^, ^3^, ^4^, ^5^ In the past JSPR survey from 2014 to 2015, a family history of psoriasis was observed in 3.9% of PsA patients, and familial onset of PsA was observed in 1.0% of the patients.^7^ In the present survey, 6.6% of the patients had a family history of psoriasis. In addition, 1.4% of patients had a family history of PsA, and there were more women (2.3%) than men (0.9%). In both cases, the most commonly affected family member was the father. Genomic imprinting might be associated with psoriasis onset in some populations.

Regarding the pathogenesis of psoriasis, various comorbidities and risk factors interact with each other.^14^ PsA is associated with metabolic syndrome and cardiovascular diseases.^15^ In the present survey, 56.3% of the patients had past history and comorbidities. The patients' medical history and comorbidities included hypertension (35.9), dyslipidemia (20.7%), diabetes mellitus (19.2%), hyperuricemia (13.5%), cardiovascular disease (4.1%), and cerebrovascular disease (3.9%). More men had comorbidities than women, and hyperuricemia was observed mostly in men (115 men [19.8%] and 7 women [2.2%]). Patients with psoriasis are less affected by lifestyle and cardiovascular diseases than those with PsA.^15^, ^16^ In contrast, PsA patients were less affected in the present survey than in the past JSPR survey of psoriasis.^5^ This finding might be partly due to the difference in severity and age distribution between the surveys.

A total of 13.7% of the patients had some type of exacerbating factors. Stress was the most common (45.5%), followed by certain seasons (31.3%) and fatigue (16.1%). Among the seasons, winter was the most commonly reported (71.4%), followed by summer (21.4%). In patients with psoriasis, stress and certain seasons were the most common exacerbating factors, and winter was the most commonly reported season in past JSPR surveys.^2^, ^3^, ^4^, ^5^ PsA and psoriasis share the same risk factors for the development of the diseases.

In the present survey, 7.3% of the patients had some type of focal infections. Odontogenic infection was the most common (41.2%), followed by tonsillitis (39.5%) and sinusitis (18.5%). In patients with psoriasis, tonsillitis was the most common focal infection in past JSPR surveys.^2^, ^3^, ^4^, ^5^ In contrast, odontogenic infection was the most common in PsA patients in the present survey.

The most common region of skin lesions at the first examination in the present survey was the scalp (57.4%), followed by the lower extremities (51.9%), back (39.1%), and upper extremities (35.7%) (Figure 4). There were no notable differences between the two sexes. In patients with psoriasis, the most common regions were the lower extremities, upper extremities, back, and scalp in the past JSPR surveys.^2^, ^3^, ^4^, ^5^ It appears that the most common region is similar between PsA and psoriasis in Japanese patients. In contrast, the most common region of joint tenderness and swelling was the finger (74.1% and 80.3%, respectively), followed by the toe (21.5% and 23.3%, respectively) (Figures 5, 6). In the present study, the peripheral type was more common than the axial type in PsA patients.

Regarding treatments, 68.6% of patients received topical therapy. Topical therapies included corticosteroids (43.7%), vitamin D_3_ (18.6%), and corticosteroid/vitamin D_3_ combinations (67.3%). The most common treatment was corticosteroid/vitamin D_3_ combinations. In contrast, 50 patients (3.0%) received phototherapy, of whom 39 and 10 patients received NB‐UVB and targeted UVB, respectively. Regarding systemic therapy, 55.9% of the patients were treated with oral medications, and 45.5% were treated with biologics. The most common oral medication was methotrexate (39.1%), followed by apremilast (27.4%). NSAIDs were also used in many patients (40.3%). Among the biologics, the most common was adalimumab (30.1%), followed by secukinumab (20.9%) and ixekizumab (17.0%). Bioswitching was observed in 13.3% of the patients, which was common among infliximab, adalimumab, secukunumab, ixekizumab, and brodalumab. In the past JSPR survey from 2014 to 2015, biologics were used in 55.5% of PsA patients, while 31% of them used disease‐modifying antirheumatic drugs (DMARDs).^7^ In the past JSPR survey of PsA in 2016, systemic therapies included biologics (52.7%), DMARDs (39.8%), and NSAIDs (31.0%).^10^ Conventional systemic therapies included methotrexate (27.2%), cyclosporin (6.3%), and sulfasalazine (4.7%). For biologics, adalimumab was the most common (41.1%), followed by infliximab (33.3%), secukinumab (13.5%), and ustekinumab (10.4%).^10^ In the past JSPR survey in 2017, bioswitching was observed in 38 out of 230 PsA cases (16.5%), and the initial biologics included infliximab (16 cases), adalimumab (11 cases), ustekinumab (5 cases), secukinumab (5 cases), and ixekizumab (1 case).^11^ Bioswitching was common among infliximab, adalimumab, and secukunumab.^11^ Regarding oral medications, apremilast became available in 2017 and methotrexate was approved for the treatment of psoriasis in Japan in 2019. Among biologics, secukinumab became available in 2015, while ixekizumab became available in 2016. Compared with the previous survey, the use of apremilast and methotrexate increased. In addition, the development of IL‐17 inhibitors has changed the treatment trend in PsA patients, and the use of infliximab has markedly decreased. However, the treatment trend for biologics did not change from 2017 to 2020 in the present survey. In contrast, only two patients (0.1%) received GMA, which was not common during the observation period.

The present survey evaluated data from annual epidemiological surveys of PsA patients from 2017 to 2020. This retrospective study did not include all Japanese patients with PsA. However, the results will provide new information regarding the recent perspective of PsA in the Japanese society. The JSPR will continue to conduct an annual epidemiological survey and additional case accumulation is anticipated in the future.

## CONFLICT OF INTEREST

M.O. has received a grant for research and/or honoraria for lectures and/or advisory membership participation from Abbvie, Celgene, Eisai, Eli Lilly, Janssen, LEO Pharma, Maruho, Mitsubishi Tanabe Pharma, Novartis, Taiho Pharmaceutical and Torii Pharmaceutical.

## Supporting information


**Table S1.** Age and sex distribution.
**Table S2.** Age at onset of skin lesions.
**Table S3.** Age at onset of joint symptoms.
**Table S4.** Anatomical distribution of skin lesions at the first examination.
**Table S5.** Anatomical distribution of joint tenderness at the first examination.
**Table S6.** Anatomical distribution of joint swelling at the first examination.
**Table S7.** Treatment trends in topical therapy.
**Table S8.** Treatment trends in phototherapy.
**Table S9.** Treatment trends in the oral medication.
**Table S10.** Treatment trends in the biologics.
