# Supplementary material for: Epidemiological survey of patients with psoriatic arthritis in the Japanese Society for Psoriasis Research from 2017 to 2020
Source: J Dermatol. 2022 Oct 19;50(1):12–25. doi: 10.1111/1346-8138.16603 (PMC10092149; doi:10.1111/1346-8138.16603)
Supplement: Supplementary file 1 — Table S1. Age and sex distribution. Table S2. Age at onset of skin lesions. Table S3. Age at onset of joint symptoms. Table S4. Anatomical distribution of skin lesions at the first examination. Table S5. Anatomical distribution of joint tenderness at the first examination. Table S6. Anatomical distribution of joint swelling at the first examination. Table S7. Treatment trends in topical therapy. Table S8. Treatment trends in phototherapy. Table S9. Treatment trends in the oral medication. Table S10. Treatment trends in the biologics. [file JDE-50-12-s001.docx]

**SUPPLEMENTAL TABLES**

**TABLE S1** Age and sex distribution.

|  | Male | Female | All |
| --- | --- | --- | --- |
| 0-9 years | 0 (0%) | 2 (0.3%) | 2 (0.1%) |
| 10-19 years | 11 (1.1%) | 9 (1.5%) | 20 (1.2%) |
| 20-29 years | 56 (5.4%) | 20 (3.3%) | 76 (4.6%) |
| 30-39 years | 133 (12.9%) | 54 (8.9%) | 187 (11.4%) |
| 40-49 years | 308 (29.8%) | 156 (25.6%) | 464 (28.3%) |
| 50-59 years | 243 (23.5%) | 208 (34.2%) | 451 (27.5%) |
| 60-69 years | 192 (18.6%) | 99 (16.3%) | 291 (17.7%) |
| 70-79 years | 74 (7.2%) | 51 (8.4%) | 125 (7.6%) |
| 80-89 years | 15 (1.5%) | 10 (1.6%) | 25 (1.5%) |
| 90- years | 0 (0%) | 0 (0%) | 0 (0%) |

**TABLE S2** Age at onset of skin lesions.

|  | Male | Female | All |
| --- | --- | --- | --- |
| 0-9 years | 5 (0.5%) | 9 (1.6%) | 14 (0.9%) |
| 10-19 years | 72 (7.4%) | 66 (11.8%) | 138 (9.0%) |
| 20-29 years | 198 (20.4%) | 97 (17.4%) | 295 (19.3%) |
| 30-39 years | 236 (24.3%) | 101 (18.1%) | 337 (22.0%) |
| 40-49 years | 211 (21.7%) | 112 (20.1%) | 323 (21.1%) |
| 50-59 years | 150 (15.4%) | 96 (17.2%) | 246 (16.1%) |
| 60-69 years | 74 (7.6%) | 56 (10.0%) | 130 (8.5%) |
| 70-79 years | 23 (2.4%) | 18 (3.2%) | 41 (2.7%) |
| 80-89 years | 2 (0.2%) | 3 (0.5%) | 5 (0.3%) |
| 90- years | 0 (0%) | 0 (0%) | 0 (0%) |

**TABLE S3** Age at onset of joint symptoms.

|  | Male | Female | All |
| --- | --- | --- | --- |
| 0-9 years | 0 (0%) | 2 (0.4%) | 2 (0.1%) |
| 10-19 years | 18 (1.9%) | 13 (2.3%) | 31 (2.1%) |
| 20-29 years | 76 (8.0%) | 36 (6.5%) | 112 (7.5%) |
| 30-39 years | 166 (17.6%) | 80 (14.4%) | 246 (16.4%) |
| 40-49 years | 287 (30.4%) | 153 (27.5%) | 440 (29.3%) |
| 50-59 years | 198 (21.0%) | 163 (29.3%) | 361 (24.0%) |
| 60-69 years | 142 (15.0%) | 74 (13.3%) | 216 (14.4%) |
| 70-79 years | 51 (5.4%) | 30 (5.4%) | 81 (5.4%) |
| 80-89 years | 7 (0.7%) | 6 (1.1%) | 13 (0.9%) |
| 90- years | 0 (0%) | 0 (0%) | 0 (0%) |

**TABLE S4** Anatomical distribution of skin lesions at the first examination.

|  | Male | Female | All |
| --- | --- | --- | --- |
| Scalp | 633 (61.3%) | 309 (50.7%) | 942 (57.4%) |
| Face | 238 (23.1%) | 84 (13.8%) | 322 (19.6%) |
| Ear | 104 (10.1%) | 75 (12.3%) | 179 (10.9%) |
| Tongue | 1 (0.1%) | 0 (0%) | 1 (0.1%) |
| Neck | 100 (9.7%) | 49 (8.0%) | 149 (9.1%) |
| Chest | 287 (27.8%) | 112 (18.4%) | 399 (24.3%) |
| Abdomen | 338 (32.8%) | 141 (23.2%) | 479 (29.2%) |
| Umbilicus | 83 (8.0%) | 28 (4.6%) | 111 (6.8%) |
| Upper extremities | 370 (35.9%) | 216 (35.5%) | 586 (35.7%) |
| Elbow | 265 (25.7%) | 148 (24.3%) | 413 (25.2%) |
| Palm | 76 (7.4%) | 39 (6.4%) | 115 (7.0%) |
| Dorsum of the hand | 151 (14.6%) | 76 (12.5%) | 227 (13.8%) |
| Finger | 192 (18.6%) | 82 (13.5%) | 274 (16.7%) |
| Fingernail | 328 (31.8%) | 143 (23.5%) | 471 (28.7%) |
| Lower extremities | 573 (55.5%) | 279 (45.8%) | 852 (51.9%) |
| Knee | 197 (19.1%) | 124 (20.4%) | 321 (19.6%) |
| Sole | 41 (4.0%) | 31 (5.1%) | 72 (4.4%) |
| Dorsum of the foot | 122 (11.8%) | 55 (9.0%) | 177 (10.8%) |
| Toe | 71 (6.9%) | 32 (5.3%) | 103 (6.3%) |
| Toenail | 145 (14.1%) | 66 (10.8%) | 211 (12.9%) |
| Back | 438 (42.4%) | 203 (33.3%) | 641 (39.1%) |
| Buttock | 315 (30.5%) | 141 (23.2%) | 456 (27.8%) |
| Gluteal cleft | 88 (8.5%) | 33 (5.4%) | 121 (7.4%) |
| Genitalia | 40 (3.9%) | 19 (3.1%) | 59 (3.6%) |
| Intertriginous area | 62 (6.0%) | 32 (5.3%) | 94 (5.7%) |

**TABLE S5** Anatomical distribution of joint tenderness at the first examination.

|  | Male | Female | All |
| --- | --- | --- | --- |
| Finger | 642 (70.9%) | 439 (79.4%) | 1,081 (74.1%) |
| Wrist | 136 (15.0%) | 96 (17.4%) | 232 (15.9%) |
| Elbow | 97 (10.7%) | 56 (10.1%) | 153 (10.5%) |
| Shoulder | 134 (14.8%) | 86 (15.6%) | 220 (15.1%) |
| Sternocostoclavicular region | 22 (2.4%) | 17 (3.1%) | 39 (2.7%) |
| Jaw | 10 (1.1%) | 2 (0.4%) | 12 (0.8%) |
| Cervical spine | 47 (5.2%) | 21 (3.8%) | 68 (4.7%) |
| Spine | 31 (3.4%) | 20 (3.6%) | 51 (3.5%) |
| Lumbar spine | 72 (8.0%) | 59 (10.7%) | 131 (9.0%) |
| Sacroiliac | 46 (5.1%) | 38 (6.9%) | 84 (5.8%) |
| Knee | 180 (19.9%) | 99 (17.9%) | 279 (19.1%) |
| Ankle | 165 (18.2%) | 99 (17.9%) | 264 (18.1%) |
| Toe | 214 (23.6%) | 99 (17.9%) | 313 (21.5%) |

**TABLE S6** Anatomical distribution of joint swelling at the first examination.

|  | Male | Female | All |
| --- | --- | --- | --- |
| Finger | 542 (77.8%) | 341 (84.8%) | 883 (80.3%) |
| Wrist | 63 (9.2%) | 43 (10.7%) | 107 (9.7%) |
| Elbow | 24 (3.4%) | 15 (3.7%) | 39 (3.5%) |
| Shoulder | 17 (2.4%) | 18 (4.5%) | 35 (3.2%) |
| Sternocostoclavicular region | 8 (1.1%) | 1 (0.2%) | 9 (0.8%) |
| Jaw | 0 (0%) | 1 (0.2%) | 1 (0.1%) |
| Cervical spine | 7 (1.0%) | 1 (0.2%) | 8 (0.7%) |
| Spine | 4 (0.6%) | 1 (0.2%) | 5 (0.5%) |
| Lumbar spine | 13 (1.9%) | 2 (0.5%) | 15 (1.4%) |
| Sacroiliac | 15 (2.2%) | 4 (1.0%) | 19 (1.7%) |
| Knee | 67 (9.6%) | 35 (8.7%) | 102 (9.3%) |
| Ankle | 90 (12.9%) | 34 (8.5%) | 124 (11.3%) |
| Toe | 180 (25.8%) | 76 (18.9%) | 256 (23.3%) |

**TABLE S7** Treatment trends in topical therapy.

|  |  | Male | Female | All |
| --- | --- | --- | --- | --- |
| 2017 | Corticosteroids | 67 (30.7%) | 49 (41.5%) | 116 (34.5%) |
|  | Vitamin D_3_ | 32 (14.7%) | 21 (17.8%) | 53 (15.8%) |
|  | Corticosteroid/Vitamin D_3_ | 91 (41.7%) | 51 (43.2%) | 142 (42.3%) |
|  | Tacrolimus | 5 (2.3%) | 0 (0%) | 5 (1.5%) |
|  | Others | 3 (1.4%) | 1 (0.8%) | 4 (1.2%) |
| 2018 | Corticosteroids | 44 (20.8%) | 39 (25.0%) | 83 (22.6%) |
|  | Vitamin D_3_ | 25 (11.8%) | 17 (10.9%) | 42 (11.4%) |
|  | Corticosteroid/Vitamin D_3_ | 91 (42.9%) | 77 (49.4%) | 168 (45.7%) |
|  | Tacrolimus | 5 (2.4%) | 0 (0%) | 5 (1.4%) |
|  | Others | 0 (0%) | 4 (2.6%) | 4 (1.1%) |
| 2019 | Corticosteroids | 88 (28.1%) | 54 (29.7%) | 142 (28.7%) |
|  | Vitamin D_3_ | 42 (13.4%) | 19 (10.4%) | 61 (12.3%) |
|  | Corticosteroid/Vitamin D_3_ | 156 (49.8%) | 86 (47.3%) | 242 (48.9%) |
|  | Tacrolimus | 4 (1.3%) | 0 (0%) | 4 (0.8%) |
|  | Others | 9 (2.9%) | 7 (3.8%) | 16 (3.2%) |
| 2020 | Corticosteroids | 103 (35.6%) | 48 (31.4%) | 151 (34.2%) |
|  | Vitamin D_3_ | 39 (13.5%) | 14 (9.2%) | 53 (12.0%) |
|  | Corticosteroid/Vitamin D_3_ | 132 (45.7%) | 74 (48.4%) | 206 (46.6%) |
|  | Tacrolimus | 6 (2.1%) | 3 (2.0%) | 9 (2.0%) |
|  | Others | 6 (2.1%) | 2 (1.3%) | 8 (1.8%) |

**TABLE S8** Treatment trends in phototherapy.

|  |  | Male | Female | All |
| --- | --- | --- | --- | --- |
| 2017 | PUVA | 0 (0%) | 0 (0%) | 0 (0%) |
|  | NB-UVB | 5 (2.3%) | 5 (4.2%) | 10 (3.0%) |
|  | BB-UVB | 1 (0.5%) | 0 (0%) | 1 (0.3%) |
|  | Targeted UVB | 1 (0.5%) | 0 (0%) | 1 (0.3%) |
| 2018 | PUVA | 0 (0%) | 0 (0%) | 0 (0%) |
|  | NB-UVB | 4 (1.9%) | 4 (2.6%) | 8 (2.2%) |
|  | BB-UVB | 0 (0%) | 0 (0%) | 0 (0%) |
|  | Targeted UVB | 3 (1.4%) | 2 (1.3%) | 5 (1.4%) |
| 2019 | PUVA | 0 (0%) | 0 (0%) | 0 (0%) |
|  | NB-UVB | 7 (2.2%) | 7 (3.8%) | 14 (2.8%) |
|  | BB-UVB | 0 (0%) | 0 (0%) | 0 (0%) |
|  | Targeted UVB | 1 (0.3%) | 1 (0.5%) | 2 (0.4%) |
| 2020 | PUVA | 0 (0%) | 0 (0%) | 0 (0%) |
|  | NB-UVB | 6 (2.1%) | 1 (0.7%) | 7 (1.6%) |
|  | BB-UVB | 0 (0%) | 0 (0%) | 0 (0%) |
|  | Targeted UVB | 1 (0.3%) | 1 (0.7%) | 2 (0.5%) |

BB-UVB, broadband ultraviolet B; NB-UVB, narrowband ultraviolet B; PUVA, psoralen ultraviolet A.

**TABLE S9** Treatment trends in the oral medication.

|  |  | Male | Female | All |
| --- | --- | --- | --- | --- |
| 2017 | NSAIDs | 41 (18.8%) | 28 (23.7%) | 69 (20.5%) |
|  | Etretinate | 9 (4.1%) | 0 (0%) | 9 (2.7%) |
|  | Methotrexate | 46 (21.1%) | 24 (20.3%) | 70 (20.8%) |
|  | Cyclosporin | 10 (4.6%) | 4 (3.4%) | 14 (4.2%) |
|  | Apremilast | 24 (11.0%) | 14 (11.9%) | 38 (11.3%) |
|  | Corticosteroids | 12 (5.5%) | 3 (2.5%) | 15 (4.5%) |
|  | Others | 15 (6.9%) | 7 (5.9%) | 22 (6.5%) |
| 2018 | NSAIDs | 35 (16.5%) | 34 (21.8%) | 69 (18.8%) |
|  | Etretinate | 2 (0.9%) | 4 (2.6%) | 6 (1.6%) |
|  | Methotrexate | 39 (18.4%) | 26 (16.7%) | 65 (17.7%) |
|  | Cyclosporin | 6 (2.8%) | 4 (2.6%) | 10 (2.7%) |
|  | Apremilast | 34 (16.0%) | 31 (19.9%) | 65 (17.7%) |
|  | Corticosteroids | 4 (1.9%) | 5 (3.2%) | 9 (2.4%) |
|  | Others | 6 (2.8%) | 15 (9.6%) | 21 (5.7%) |
| 2019 | NSAIDs | 75 (24.0%) | 42 (23.1%) | 117 (23.6%) |
|  | Etretinate | 2 (0.6%) | 3 (1.6%) | 5 (1.0%) |
|  | Methotrexate | 81 (25.9%) | 38 (20.9%) | 119 (24.0%) |
|  | Cyclosporin | 5 (1.6%) | 6 (3.3%) | 11 (2.2%) |
|  | Apremilast | 41 (13.1%) | 29 (15.9%) | 70 (14.1%) |
|  | Corticosteroids | 12 (3.8%) | 5 (2.7%) | 17 (3.4%) |
|  | Others | 30 (9.6%) | 15 (8.2%) | 45 (9.1%) |
| 2020 | NSAIDs | 65 (22.5%) | 50 (32.7%) | 115 (26.0%) |
|  | Etretinate | 5 (1.7%) | 0 (0%) | 5 (1.1%) |
|  | Methotrexate | 66 (22.8%) | 39 (25.5%) | 105 (23.8%) |
|  | Cyclosporin | 3 (1.0%) | 3 (2.0%) | 6 (1.4%) |
|  | Apremilast | 52 (18.0%) | 26 (17.0%) | 78 (17.6%) |
|  | Corticosteroids | 8 (2.8%) | 5 (3.3%) | 13 (2.9%) |
|  | Others | 16 (5.5%) | 12 (7.8%) | 28 (6.3%) |

NSAIDs: non-steroidal anti-inflammatory drugs.

**TABLE S10** Treatment trends in the biologics.

|  |  | Male | Female | All |
| --- | --- | --- | --- | --- |
| 2017 | Infliximab | 17 (7.8%) | 3 (2.5%) | 20 (6.0%) |
|  | Adalimumab | 46 (21.1%) | 35 (29.7%) | 81 (24.1%) |
|  | Certolizumab pegol | 1 (0.5%) | 0 (0%) | 1 (0.3%) |
|  | Ustekinumab | 5 (2.3%) | 2 (1.7%) | 7 (2.1%) |
|  | Secukinumab | 12 (5.5%) | 7 (5.9%) | 19 (5.7%) |
|  | Ixekizumab | 17 (7.8%) | 8 (6.8%) | 25 (7.4%) |
|  | Brodalumab | 11 (5.0%) | 4 (3.4%) | 15 (4.5%) |
|  | Guselkumab | 2 (0.9%) | 2 (1.7%) | 4 (1.2%) |
|  | Risankizumab | 0 (0%) | 0 (0%) | 0 (0%) |
|  | Tildrakizumab | 0 (0%) | 0 (0%) | 0 (0%) |
|  | Biosimilar | 0 (0%) | 0 (0%) | 0 (0%) |
|  | Others | 1 (0.5%) | 1 (0.8%) | 2 (0.6%) |
| 2018 | Infliximab | 18 (8.5%) | 5 (3.2%) | 23 (6.3%) |
|  | Adalimumab | 31 (14.6%) | 17 (10.9%) | 48 (13.0%) |
|  | Certolizumab pegol | 0 (0%) | 0 (0%) | 0 (0%) |
|  | Ustekinumab | 3 (1.4%) | 5 (3.2%) | 8 (2.2%) |
|  | Secukinumab | 16 (7.5%) | 12 (7.7%) | 28 (7.6%) |
|  | Ixekizumab | 21 (9.9%) | 8 (5.1%) | 29 (7.9%) |
|  | Brodalumab | 1 (0.5%) | 3 (1.9%) | 4 (1.1%) |
|  | Guselkumab | 7 (3.3%) | 7 (4.5%) | 14 (3.8%) |
|  | Risankizumab | 0 (0%) | 0 (0%) | 0 (0%) |
|  | Tildrakizumab | 0 (0%) | 0 (0%) | 0 (0%) |
|  | Biosimilar | 0 (0%) | 0 (0%) | 0 (0%) |
|  | Others | 5 (2.4%) | 5 (3.2%) | 10 (2.7%) |
| 2019 | Infliximab | 7 (2.2%) | 3 (1.6%) | 10 (2.0%) |
|  | Adalimumab | 44 (14.1%) | 17 (9.3%) | 61 (12.3%) |
|  | Certolizumab pegol | 4 (1.3%) | 2 (1.1%) | 6 (1.2%) |
|  | Ustekinumab | 1 (0.3%) | 1 (0.5%) | 2 (0.4%) |
|  | Secukinumab | 35 (11.2%) | 21 (11.5%) | 56 (11.3%) |
|  | Ixekizumab | 21 (6.7%) | 19 (10.4%) | 40 (8.1%) |
|  | Brodalumab | 5 (1.6%) | 2 (1.1%) | 7 (1.4%) |
|  | Guselkumab | 9 (2.9%) | 9 (4.9%) | 18 (3.6%) |
|  | Risankizumab | 10 (3.2%) | 4 (2.2%) | 14 (2.8%) |
|  | Tildrakizumab | 0 (0%) | 0 (0%) | 0 (0%) |
|  | Biosimilar | 1 (0.3%) | 0 (0%) | 1 (0.2%) |
|  | Others | 1 (0.3%) | 2 (1.1%) | 3 (0.6%) |
| 2020 | Infliximab | 5 (1.7%) | 0 (0%) | 5 (1.1%) |
|  | Adalimumab | 20 (6.9%) | 15 (9.8%) | 35 (7.9%) |
|  | Certolizumab pegol | 15 (5.2%) | 6 (3.9%) | 21 (4.8%) |
|  | Ustekinumab | 1 (0.3%) | 2 (1.3%) | 3 (0.7%) |
|  | Secukinumab | 33 (11.4%) | 20 (13.1%) | 53 (12.0%) |
|  | Ixekizumab | 26 (9.0%) | 7 (4.6%) | 33 (7.5%) |
|  | Brodalumab | 5 (1.7%) | 2 (1.3%) | 7 (1.6%) |
|  | Guselkumab | 6 (2.1%) | 7 (4.6%) | 13 (2.9%) |
|  | Risankizumab | 14 (4.8%) | 4 (2.6%) | 18 (4.1%) |
|  | Tildrakizumab | 1 (0.3%) | 1 (0.7%) | 2 (0.5%) |
|  | Biosimilar | 0 (0%) | 0 (0%) | 0 (0%) |
|  | Others | 1 (0.3%) | 0 (0%) | 1 (0.2%) |
